# Genome Wide Analysis for Growth at Two Growth Stages in A New Fast-Growing Common Carp Strain (*Cyprinus carpio* L.)

**DOI:** 10.1038/s41598-020-64037-w

**Published:** 2020-04-29

**Authors:** Shengyan Su, Bouzoualegh Raouf, Xinjin He, Nana Cai, Xinyuan Li, Juhua Yu, JianLin Li, Fan Yu, Meiyao Wang, Yongkai Tang

**Affiliations:** 1 Key Laboratory of Freshwater Fisheries and Germplasm Resources Utilization, Ministry of Agriculture; Freshwater Fisheries Research Center, Chinese Academy of Fishery Sciences, Wuxi, 214081 PR China; 2 Wuxi Fisheries College, Nanjing Agricultural University, Wuxi, 214081 PR China; 3 College of Animal science, Shanxi Agricultural University, Taigu, PR China

**Keywords:** Animal breeding, Animal breeding, Genetic association study, Genetic association study

## Abstract

In order to identify candidate genes or loci associated with growth performance of the newly established common carp strain, Xinlong, we conducted a genome-wide association analysis using 2b-RAD technology on 123 individuals. We constructed two sets of libraries associated with growth-related parameters (weight, length, width and depth) measured at two different grow-out stages. Among the 413,059 SNPs identified using SOAP SNP calling, 147,131 were tested for GWAS after quality filtering. Finally, 39 overlapping SNPs, assigned to four genomic locations, were associated with growth traits in two stages. These loci were assigned to functional classes related to immune response, response to stress, neurogenesis, cholesterol metabolism and development, and proliferation and differentiation of cells. By overlapping results of Plink and EMMAX analyses, we identified three genes: *TOX, PLK2* and *CD163* (both methods *P* < 0.05). Our study results could be used for marker-assisted selection to further improve the growth of the Xinlong strain, and illustrate that largely different sets of genes drive the growth of carp in the early and late grow-out stages.

## Introduction

Genome-wide association studies facilitate identification of single-nucleotide polymorphisms (SNPs) and genes associated with important economic traits. In particular, growth is one of the most economically important traits for the aquaculture industry. At the genomic level, genes and loci controlling this quantitative trait received a lot of interest in fish; for example growth rate in Atlantic salmon and rainbow trout^1–3^, head size in catfish and common carp^4,5^, etc. As a powerful statistical tool for connecting traits to their corresponding genes, genome-wide association study (GWAS) offers the possibility to analyze a massive amount of data. This allows identification of single nucleotide polymorphisms (SNP) or genes that may be related to important economic traits, or other traits of interest^6^. However, GWAS employs methods that require genome-wide SNP data produced by genome re-sequencing, so it is relatively costly, which limits its applicability^7^. Although the genechip used to capture the genome wide molecular markers has been established for some farmed animals (e.g. cattle and pigs), the application of GWAS in aquatic animals still faces higher costs and practical problems. To account for this, a simplified and cheaper genotyping method, 2b-RAD, was developed^8^. This method is based on the sequencing of uniform DNA fragments produced by type IIB restriction endonuclease. Its effectiveness in associated analysis and genotyping has been established in several freshwater and marine fish species, including bighead carp (*Hypophthalmichthys nobilis*), Nile tilapia (*Oreochromis niloticus*), and yellowfin tuna (*Thunnus albacares*)^9–12^. The common carp (*Cyprinus carpio* L.) is one of the oldest and most important farmed fish species, and the most widely distributed freshwater fish in the world^13,14^. Ranking third among the most commercially important fish species in China, its production exceeded 349,800 tons in 2016^15^. With such importance, common carp has been a subject of several breeding programs. A carp strain named “Huanghe” has a long tradition and historical importance in Chinese culture, and retains notable economic importance. As a result, despite the fact that its growth rate is not as fast as that of more modern strains^16,17^, it has received a lot of interest from researchers. Recently, a new derivative of the Huanghe strain, provisionally named “Xinlong”, has been established using a combination of the best linear unbiased prediction (BLUP) with marker-assisted technology. After six years of continuous selection, its growth rate improvement (compared to the Huanghe strain) was 20.84% after an 8 months-long growth trial^16^. In order to explore the genetic basis for this fast-growing phenotype, we previously conducted a genome-wide association study using 2b-RAD sequence assay for four growth-related traits at 3 and 8 months of age, and found that genes contributing to this improvement are related to sex, neural pathways and fatty acid metabolism^18^. However, for the aquaculture industry purposes, growth performance at the end of the culture period, i.e. when fish reach the marketable size (17 months after stocking for carp) is more important than growth rate in the middle of that period. Furthermore, it is also important to know whether growth rate is controlled by the same set of genes during the entire grow-out period, or whether different genes may be controlling growth rates in different growth stages.

In this study, we set out to identify genes driving the growth performance during the second half of the grow-out period (8–17 months). We also determined genes and loci that exhibited a strong correlation with improved growth parameters during both grow-out stages. To achieve this, we conducted a genome-wide comparative analysis of growth parameters-associated loci using the cost-effective 2b-RAD method. This allowed us to discover thousands of SNPs associated with growth performance in two different growth periods, as well as identify those overlapping between the two stages. These results shall help us better understand the genetic basis for the improved growth rate of the new Xinlong strain.

## Results

### Evaluation of growth-related traits

**Figure 1:**
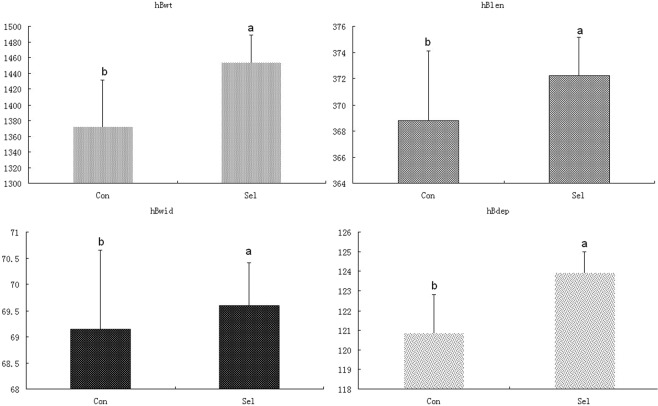
Comparison of mean growth rates of two carp populations at harvest. Con; Control group; Sel: selection group; hBwt: body weight (g); hBlen: body length (cm); hBwid: body width (cm); hBdep: body depth (cm). Data are expressed as the mean ± SD.

We analyzed four growth parameters in two different grow-out stages: five months after tagging, and at the end of the production cycle (17 months). Within-stage comparisons revealed a statistically significant linear relationship between the four growth traits. Notably, we observed particularly strong correlation between Blen5m/Bwid5m (r = 0.94) and Blen5m/Bdep5m (r = 0.91) in the first stage, and between hBwt and hBdep (r = 0.93) in the second stage (Table 1). At the end of the growth cycle, all four growth parameters were statistically significantly higher in the Selection group, whereas control group exhibited higher SD values (Fig. 1).

**Table 1:** Correlation coefficients between four growth traits of Xinlong strain at tagging and harvesting time-points.

	Blen5m	Bwid5m	Bdep5m	hBwt	hBlen	hBwid	hBdep
Bwt5m	0.74^*^	0.68^*^	0.71^*^	0.04	0.01	0.03	0.09
Blen5m	1	0.94^*^	0.91^*^	0.23^*^	0.21^*^	0.20^*^	0.25^*^
Bwid5m		1	0.91^*^	0.24^*^	0.20^*^	0.25^*^	0.27^*^
Bdep5m			1	0.24^*^	0.18	0.26^*^	0.28^*^
hBwt				1	0.85^*^	0.85^*^	0.93^*^
hBlen					1	0.71^*^	0.78^*^
hBwid						1	0.81^*^

*means P < 0.05

### 2b-RAD raw data processing

Raw 2b-RAD data were filtered via several quality control steps (see methods for details), clean data were then mapped to the genome of domesticated common carp^19^, and 413,059 SNPs detected after SNP calling by RADtyping program. After the SNP quality checking (QC), 147,131 SNPs were left and successfully assigned to 50 chromosomes and 3,377 scaffolds of the draft genome. The average sequencing depth was 44.56 and mapping successfulness rate ranged from 46.89% to 50.83%. We obtained 27,730,499 unique tags (225,451 tags per specimen) (Supplementary Table S1). Five regions accounted for 96.01% of all SNPs; with the highest proportion annotated in the intron (34.00%), followed by the intergenic (27.18%), upstream genes in the annotation file of reference genome (13.37%), downstream genes (13.18%), and finally exon regions (8.28%) (Supplementary Table S2).

### Exploring genomic markers associated with growth traits in two grow-out stages

Analyses with Plink and EMMAX were conducted for candidate SNPs associated with the four growth performance traits in each stage: 44,306 SNPs were associated with growth traits in the first stage and 33,039 in the second stage (*P* < 0.05). After the Bonferroni correction, statistically significant values (*P* < 0.05) were found for only 109 SNPs in the first stage and only one in the second stage (Table 2).

**Table 2:** Genome-wide association analysis of SNPs associated with four growth traits at tagging, 8 months, and harvest.

Growth traits	*P* < 0.05	*P* < 0.05 after Bonferroni correction
iBlen	7199	1
iBWt	7322	0
Bwt5m	7044	116
Blen5m	7429	11
Bwid5m	8329	2
Bdep5m	7957	6
hBwt	6735	1
hBlen	6704	0
hBwid	5931	0
hBdep	6592	0

Manhattan graphs plotted for parameters of all growth stages [−log10 (P value)> 5] allowed us to isolate 345 related SNPs. Using EMMAX software, which can account for full sib information, we identified 39 SNPs that were associated with the studied growth parameters via comparison of 45 (10 traits * 9/2 combination pairs) pairs of growth traits in both grow-out stages (Table 3). No overlapping loci were identified in pairwise comparisons of loci associated with growth parameters between first and second stages. These 39 SNPs were annotated using the reference genome (Table 4). Among these 39 SNPs, 36 were identified only by EMMAX, and three were further corroborated by plink analysis. These 39 loci were located in the 5' flank regions, 3' flank regions, CDS and intron regions of corresponding genes, and belonged to the following five functional classes:

1. Immune response to pathogens. In response to the microbial infection, interferon alpha/beta receptor 2 (*IFNA/betaR2*) triggers a complex cascade of events^20^. Many genes associated with these downstream events are involved in cellular immune processes. For T-cell development and trafficking, thymocyte selection-associated high mobility protein (*TOX*) is involved in chromatin assembly, transcription, and modulation of T-cell development, and growth regulation^21^. DCs and T cells in semaphorin-4A (*Sema4a*)-knockout mice displayed poor allostimulatory activities and T helper cell (Th) differentiation, respectively^22^. Spinster homolog 2 (*SPNS2)* regulates the levels of Sphingosine-1-phosphate (*S1P*) gradient that controls lymphocyte trafficking^23^. In addition, dnaJ homolog subfamily C member 25 (*DNAJC25*) can significantly increase cell apoptosis, and its overexpression inhibits cell growth^24^. Deletion of inositol polyphosphate multikinase (*IPMK*) in cell lines and mice virtually abolished lipophagy, caused liver damage and inflammation, and impaired hepatocyte regeneration^25^. Among the regulation factors, laccase domain-containing protein 1 (*LACC1*) regulates TNF and IL-17 in mouse models of arthritis and inflammation^26^. A knockout of scavenger receptor cysteine-rich type 1 protein M130 (*CD163*) improved the resistance to viral infection in pigs^27^. A knockout of apolipoprotein A-I (*APOA1*) decreased parenchymal and vascular β-amyloid pathology in the Tg2576 mouse model of Alzheimer’s disease^28^.
2. Development, proliferation and differentiation of cells. In the embryonic development of mice, tyrosine-protein kinase *JAK1* deficiency causes arrested development, and apnea and death in newborn pups^29^. In the cell proliferation and differentiation category, nudC domain-containing protein 1 (*NUDCd1*) expression was positively correlated with cell proliferation, migration, and invasion in A498 cells^30^. Compared to *smarcb1a NUDCd1* function, SWI/SNF-related, matrix-associated, actin-dependent regulator of chromatin, subfamily B, member 1-A (*smarcb1a*) is a critical component of the mammalian SWItch/Sucrose Non-Fermentable (mSWI/SNF) protein, which promotes MyoD-mediated muscle differentiation by altering the chromatin structure in promoter regions of endogenous loci^31^. APC membrane recruitment protein 1 (*Amer1*) acts as a scaffold protein for the β-catenin destruction complex and promotes stabilization of Axin at the plasma membrane, thereby exerting negative regulatory role in Wnt signaling (key role in embryonic development)^32^. Complete loss of DNA damage checkpoint protein *RAD9A* in male mice caused a radical loss of spermatogenic cells (infertility or sub-fertility)^33^.
3. Cholesterol metabolism. Phosphatidylinositol 5-phosphate 4-kinase type-2 alpha (*PIP4K2A*) regulates intracellular cholesterol transport^34^. As a result, hormone-sensitive lipase (*HSL*) has negative correlation with cholesterol content in the testes^35^, and dela(24)-sterol reductase (*DHCR24*) knockout in brain caused cholesterol deficiency in mice^36^.
4. Neurogenesis. The genes involved in this category can be divided into two groups, one of which is related to the neurons and neuritogenesis. Rotein kinase C-binding protein (*NELL2*) promotes survival of neurons^37^. Homeobox protein (*DBX1-B*) is crucial for production of dorsal habenular neurons^38^. Synapse differentiation-inducing gene protein 1 (*SYNDIG1*) regulates the excitatory synapse maturation in rats^39^. Mice lacking *Nrxn2α* (α-variant of *NRXN2* (neurexin 2)) exhibit behavioral abnormalities (social interaction deficits and increased anxiety)^40^. The other group is associated with signal transmission and regulation factors. Septin 8 B (*sept8b*) expression pattern in glial cells of zebrafish indicated that it was involved in signal transmission in nervous systems^41^. *Mtss1* impinges on directional persistence and neuritogenesis^42^. Rap guanine nucleotide exchange factor 6(*Rapgef6*)-knockout mice exhibited mild behavioral abnormalities (hyperlocomotion and working-memory defects)^43^.
5. Response to stress, comprising genes included in the interaction between environment and genetics. Catechol-O-methyltransferase (*COMT*) was identified as gene × environment interaction candidate gene (probably a regulatory factor), associated with childhood adversity, posttraumatic stress disorder, and major depressive disorder^44^. Mixed lineage kinase dual leucine zipper kinase (*DLK*) regulates the JNK-based stress response pathway^45^. Overexpression of *IMPACT* in yeast cells inhibited growth under all stress conditions that require *GCN2* (eIF-2-alpha kinase) and *GCN1* for cell survival, probably by the IMPACT promoting the dissolution of the GCN2–GCN1 complex^46^. Serine/threonine-protein kinase PLK2-like (*PLK2-like*) is involved in catalysis of the following reaction in response to stress: ATP + protein serine = ADP + protein serine phosphate, and ATP + protein threonine = ADP + protein threonine phosphate^47^.

**Table 3:** Number of SNPs identified by GWAS for ten growth parameters and for SNPs overlapping between pairs of parameters.

	Trait	Number of SNPs observed by GWAS(***P*** < **10e-5**)		
Single	iBlen	11		
	iBWt	2		
	Bwt5m	269		
	Blen5m	45		
	Bwid5m	23		
	Bdep5m	24		
	hBwt	5		
	hBlen	1		
	hBwid	4		
	hBdep	7		
Overlapping region	Pairwise overlap	SNPs of 1^st^ trait	SNPs of 2^nd^ trait	SNPs overlapped
	iBWt_ iBlen	2	11	1
	Blen5m _ Bdep5m	45	24	17
	Blen5m _ Bwid5m	45	23	22
	Blen5m _ Bwt5m	45	269	6
	Bwid5m _ Bwt5m	23	269	4
	Bdep5m _ Bwid5m	24	23	11
	hBdep _ hBwt	7	5	2
	hBwt_ hBlen	5	1	1
	hBwt_ hBwid	5	4	2
	hBdep _ hBwid	7	4	1

**Table 4:** Annotation of overlapping SNPs.

Scaffold	Locus	Gene	Location
NC_031701.1	10847582	ADP-dependent glucokinase-like	5 flanking region
NC_031705.1	10039933	protein spinster homolog 2-like	CDS
NC_031706.1	4058596	APC membrane recruitment protein 1-like	CDS
NC_031712.1	2621531	cell cycle checkpoint control protein RAD9A-like	intron
NC_031712.1	5706113	catechol O-methyltransferase-like	5 flanking region
NC_031712.1	13964866	LOC109094936	intron
NC_031713.1	11967224	Septin-8-B, transcript variant X1	5 flanking region
NC_031726.1	7049273	synapse differentiation-inducing gene protein 1-like	5 flanking region
NC_031728.1	12629280	hormone-sensitive lipase-like, transcript variant X1	CDS
NC_031728.1	2113842	interferon alpha/beta receptor 2-like	CDS
NC_031728.1	1956966	Rap guanine nucleotide exchange factor 6-like	intron
NC_031729.1	14358583	phosphatidylinositol 5-phosphate 4-kinase type-2 alpha-like	5 flanking region
NC_031729.1	8572673	tyrosine-protein kinase JAK1-like	5 flanking region
NC_031731.1	16563967	MTSS1-like protein	intron
NC_031734.1	3841135	LOC109112089	5 flanking region
NW_017537781.1	436187	mitogen-activated protein kinase 12	5 flanking region
NW_017537915.1	612379	laccase domain-containing protein 1-like	5 flanking region
NW_017537915.1	612367	laccase domain-containing protein 1-like	5 flanking region
NW_017537926.1	388773	nudC domain-containing protein 1	CDS
NW_017537954.1	30977	delta(24)-sterol reductase-like	3 flanking region
NW_017538012.1	689985	inositol polyphosphate multikinase-like	intron
NW_017538056.1	231523	uncharacterized LOC109058697	3 flanking region
NW_017538109.1	569817	thymocyte selection-associated high mobility group box protein TOX-like	5 flanking region
NW_017538218.1	113607	uncharacterized LOC109062889	5 flanking region
NW_017539969.1	119751	eIF-2-alpha kinase activator GCN1-like	CDS
NW_017540424.1	362678	neurexin-2-like	5 flanking region
NW_017540424.1	362692	neurexin-2-like	5 flanking region
NW_017540444.1	157894	apolipoprotein A-I	intron
NW_017541285.1	11708	protein kinase C-binding protein NELL2-like	5 flanking region
NW_017541427.1	19670	scavenger receptor cysteine-rich type 1 protein M130-like	intron
NW_017538165.1	106199	Serine/threonine-protein kinase PLK2-like	CDS
NW_017541589.1	125406	semaphorin-4A-like	intron
NW_017541589.1	125410	semaphorin-4A-like	intron
NW_017542184.1	50564	uncharacterized LOC109080123	3 flanking region
NW_017542350.1	76956	dnaJ homolog subfamily C member 25-like	3 flanking region
NW_017542510.1	28930	SWI/SNF-related matrix-associated actin-dependent regulator of chromatin subfamily B member 1-A-like	5 flanking region
NW_017543266.1	127635	uncharacterized LOC109083918	3 flanking region
NW_017543773.1	1097335	uncharacterized LOC109086122	3 flanking region
NW_017545334.1	380834	homeobox protein DBX1-B	CDS

We calculated pairwise R^2^ values between each of the three common SNPs associated with growth traits and other SNPs identified on the same chromosome or scaffold, and selected all pairs with R^2^ > 0.1. Manhattan plots for Blen5m (NW_017538109.1–569817 and NW_017541427.1–19670) and Bwid5m (W_017538165.1–106199 and NW_017541427.1–19670) are shown in Figures S1 and S2, and linkage disequilibrium for the three SNPs in Figure S3. Selected pairs were located on the position 521711 (annotation: exon of mitochondrial ribosomal protein L57 (*MRPL57*), transcript variant X1) in scaffold NW_017538109.1 (R^2^ = 0.11), position 396390 (annotation: intron of 1-acyl-sn-glycerol-3-phosphate acyltransferase epsilon-like (*AGPAT5*)) in scaffold NW_017538165.1 (R2 = 0.12), and position 65778 (annotation: uncharacterized LOC109077101) in scaffold NW_017541427.1 (R^2^ = 0.44).

### Validation of growth-related markers

**Figure 2:**
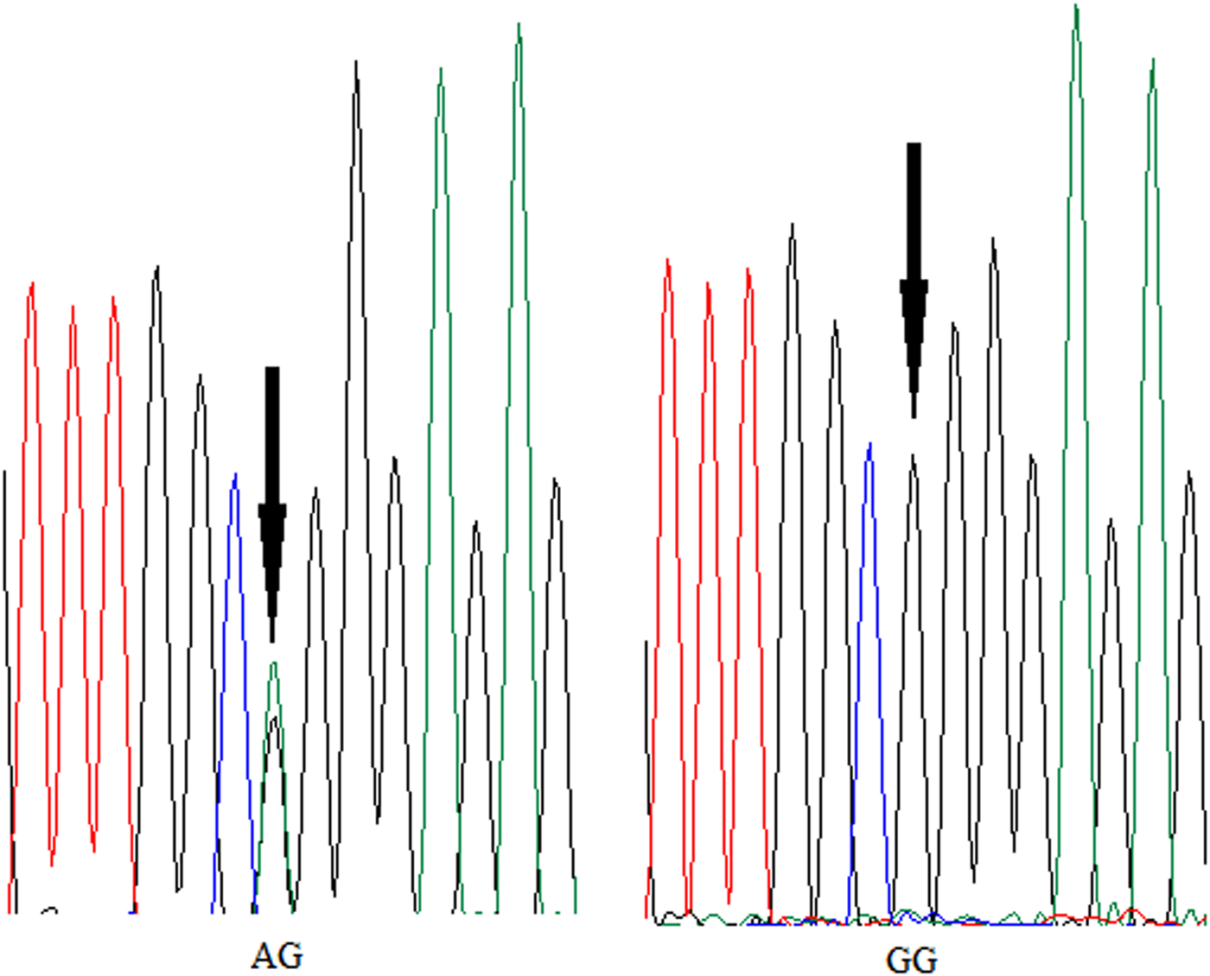
Effect of *PLK2* genotype observed by GWAS on four growth parameters of the Xinlong carp strain after 8 months of grow-out culture. (**a**) Bwt5m (body weight, g), Blen5m (body length, mm), Bdep5m (body depth, mm) and BWid5m (body width, mm). Different characters represent significant differences between two genotypes. (**b**) DNA sequencing electropherograms of the AG and GG genotypes. The targeted SNP sites are indicated by black arrows.

To verify the authenticity of the three genes (*TOX*, *PLK2 and CD163*) identified by overlapping the results of Plink and EMMAX analyses in 188 specimens, three primer pairs were designed to amplify and sequence the PCR products. Among the sequenced products, two *PLK2* genotypes, AG and GG, were identified. We found that two genotypes exhibited significant differences in the BWid5m growth parameter (Fig. 2).

## Discussion

Genome-wide association studies are a very efficient approach for associating SNPs with important economic traits, as they allow analysis of large amounts of SNP data and identification of specific markers^6,48^. Genomic selection based on specific genes can be a cost-effective and fast (regarding the number of genome-wide markers used) method to considerably facilitate the selection for complex quantitative traits by precisely selecting favourable alleles. Obtaining thousands of SNP markers is becoming easier with the development of new technologies for population genotyping, thus allowing construction of high-density genetic maps for domesticated animals, as well as identification of QTL and specific markers for economically important traits^49^. High and low-density genome-wide association assays have been constructed both for terrestrial and aquatic farmed animals. Examples include the bovine SNP50 chip with 54,001 SNPs^50^, the SNP linkage map developed for Atlantic salmon, with around 5500 markers resulting from 16.5 K SNPs, and the SNP linkage map of common carp with 28,194 SNPs from a 250 K genechip^51,52^. These SNP arrays were used to explore QTL related to important economic traits, such as body weight and age at sexual maturation^4^. Xu *et al*. relied on QTL mapping to identify a number of growth performance regulators, including those associated with cell-proliferation (*IGF1*) and growth and energy metabolism (*ERBB4*, *BMPR1B*, *SMTLB*, *GS*, *KISS2* and *NPFFR1*)^52^. In our previous genome-wide analysis of the Xinlong strain, we found a number of genes associated with growth parameters at the early grow-out stage, including those related to neural pathways (*RHEB*, *MDGA2*, *NRXN1a*, *BDNF*, *SHANK2* and *REEP2*), sex dimorphism (*NRXN1A*, *SMOC2*, *RAB10* and *HSD17B*), and fatty acid metabolism (*RAB10*, *TNMD*, *VPAC2* and *ACSF2*)^18^. However, it remained unknown which candidate genes or loci contribute to the growth performance during later growth stages.

In this study, we were interested in genes controlling the growth parameters in later grow-out stages, up until the marketable fish size. By studying ten growth traits in two different grow-out stages, and then merging the results of Plink and EMMAX analyses, we identified 39 growth-related SNPs. SNPs associated with five functional classes were associated with four growth parameters in the first grow-out stage. No overlapping SNPs were found between growth performance parameters in the first and second grow-out stages. This may be a genetic molecular marker contribution to the comparatively high correlation coefficients among the former four parameters and lower coefficients between the growth parameters in the two grow-out stages (Table 1). These overlapping genes may be interacting in a way that produces the observed increased growth performance. Firstly, both *HSL* (regulates the cholesterol content^35^) and *PIP4K2A* (regulates the intracellular cholesterol transport^34^) can influence the availability of cholesterol. The cholesterol level appears to limit development of the central nervous system (CNS)^36,53^. The LENSSPQAPARRLLPP (BigLEN) neuropeptide delivered to synapse is believed to act through the G protein-coupled receptor 171 (*GPR171*) to regulate body weight by food intake and metabolism^54^. Beside the body weight, hypothalamic neurons may be involved in the regulation of metabolism, energy balance, and social behavior^55^. Within the brain, endogenous leptin plays a physiologically important role in the control of food intake^56^. In the neural cell lines, leptin-induced transactivation of *NPY* gene (involved in the central and sympathetic regulation of food intake) promoter can be mediated by *JAK1*^57^. This gene was singled out in our pairwise comparisons of growth parameters, and six other genes were involved in pathways associated with neurons and neurogenesis (*NELL2*, *dbx1b*, *Mtss1*, *SYNDIG1*, *NRXN2* and *BDNF*). Thus, cholesterol content and transportation can affect the central nervous system and the brain, both of which play a role in the control of food intake via *leptin*, *JAK1* and *GPR171* genes.

By overlapping Plink and EMMAX results, we identified three genes. *TOX* is required for a critical transitional step in selection and maturation of thymocytes, so this gene may have a direct effect on the cell-mediated immunity^58^. Therefore, we speculate that this gene may play a role in immune responses and growth of the common carp. *PLK2*-like is associated with metabolic responses to stress, involved in cell proliferation by contributing to the mitosis and centrosome cycle, and plays an important role in embryonic and skeletal development, as evidenced by experiments on cultured *PLK2* embryonic fibroblasts, where the proliferation was stronger in cells expressing *PLK2*^59,60^. And finally, *CD163*-knockout pigs were completely resistant to viral infection during the PRRSV challenge due to the fact that this gene acts as a cellular receptor for PRRSV^27^.

By LD analysis of three common loci, two candidate genes were found: *AGPAT5* and *MRPL57*. Polymorphism of *AGPAT5* gene was associated with the pig and cattle meat quality^61,62^. Liver-specific Agpat5 knockout mice had significantly reduced fasting plasma insulin and hepatic triglycerides after 12 weeks of high-fat diet^63^. AGPAT2 enzyme catalyzes the acylation of lysophosphatidic acid to form phosphatidic acid, a key intermediate in the biosynthesis of triacylglycerol and glycerophospholipids^64^. Therefore, regulation of biosynthesis of triacylglycerol and glycerophospholipids may be the genetic explanaiton behind higher content of polyunsaturated fatty acids in the muscle of the Jinlong carp^18^.

The candidate genes explored in this paper indicate that growth of common carp is a complex trait, associated with immunity, metabolism, stress and development, and contingent upon the interaction between environment and genetic backgroud^65^. Therefore, genome-based selective breeding should not only focus on traditional growth-related genes, but also use reliable breeding population to explore additional genes that may affect growth. As many of these genes remain unknown for the time being, it may be a promising direction for future studies.

## Conclusions

The objective of this study was to use the cost-effective 2b-RAD technology to investigate the genomic regions related to the growth performance of a new common carp strain. Growth-related parameters, measured at 8 months of age and at the end of the grow-out cycle, were evaluated for 123 specimens. According to these results, we analyzed 2b-RAD data and identified a total of 147,131 SNPs. A comparison of loci associated with all growth parameters in the first and second grow-out stages identified 39 SNPs. None of these SNP overlapped between the first grow-out stage and second grow-out stage. Genes associated with these SNPs belonged to the following five functional classes: immune response to pathogens, development, proliferation and differentiation of cells, cholesterol metabolism, neurogenesis and response to stress. Plink and EMMAX analyzes singled-out three genes: *TOX, PLK2* and *CD163*.

## Methods

### Experimental material and sampling

Focusing on growth performance as the target trait, a genetically distinct strain was developed from the traditional Huanghe carp strain at the Freshwater Fisheries Research Center, Chinese Academy of Fishery Sciences using BLUP^66^ method according to the best-predicted breeding values. A population of the new strain was obtained using artificial breeding (spawning in females was stimulated with hormonal injections, and then roe and milt mixed manually), fertilized eggs were incubated in separate cage settle nets for one week, and larvae then transferred to labelled (ID + spawning time) nursery happas. After 3 months in the nursery, parents and 8 of their full-sib F1 progenies randomly selected from each family were anesthetized with clove oil (75 mg/L)^67^ and tagged with passive integrated transponder tags (PIT) produced by Biomark. In total, 123 tagged full-sib common carp progeny specimens belonging to 25 families were selected for the experiment. Xinlong strain was represented by 95 specimens belonging to 19 families, and the control population (Huanghe strain) by 28 specimens belonging to 6 families. These two populations were cultivated in concrete tanks supplied with aerated and filtered water. During 17 months of the culture period, counting from the date of tagging (3 months of age) to the harvesting time (20 months of age), the fish were fed with a commercial feed (30% protein content) at a daily dose of 5% of their approximate body weight. Physicochemical parameters of the water were regularly monitored: dissolved oxygen, pH and temperature.

Growth parameters were measured after 5 (5 m) and 17 (h) months of the growth trial: body weight (Bwt5m and hBwt), body length (Blen5m and hBlen), body depth (Bdep5m and hBdep) and finally body width (BWid5m and hBWid), respectively. Fish were anesthetized with clove oil (75 mg·l^−1^) during the measurement procedure. Between 50 and 100 mg of caudal fin tissue was collected from each individual at the harvesting stage and preserved in 95% alcohol at 4 °C until DNA extraction.

Experimental procedures and animal handling were carried out in accordance with guidelines for the care and use of animals for scientific purposes set by the Institutional Animal Care and Use Committee of the Freshwater Fisheries Research Center and approved by the animal ethics committee of Chinese Academy of Fishery Sciences.

### DNA extraction and 2b-RAD sequencing

DNA was isolated using universal genomic DNA kit (CWBIO, China) following the manual. Briefly, around 25 mg of fin tissue was digested for 1 hour at 56 °C with 20 µl Proteinase K to remove the proteins, and then incubated for 10 minutes at 70 °C after adding Buffer GL and 100% ethanol. Once the solutions were transferred to a column with a collection tube (Spin Columns DM), they were processed with Buffer GW1 and Buffer GW2 in order to improve the DNA purity. Finally, Buffer GE was added and DNA precipitated, collected and stored at −20 °C. Quality and concentration of the extracted DNA were checked by spectrophotometry (optical density reading at 260 and 280 nm) and electrophoresis on 1.0% agarose gel.

In order to construct the 2b-RAD libraries for each of the 123 individuals, we followed a simplified RAD (restriction site–associated DNA) genotyping method, as described by Su *et al*.^18^, with minor modifications. This method is based on sequencing of uniform fragments produced by type IIB restriction endonucleases^8^, as described in the 2b-RAD protocol^3^. PCR products were purified and quantified using SPRI select purification kit (Beckman Coulter, Pasadena, CA, USA) and Qubit 2.0 fluorometer (Invitrogen). The quality of all amplicon libraries was checked on 1.8% agarose gels and verified on Agilent 2100 Bioanalyzer.

### 2b-RAD raw data processing

In order to filter the sequenced reads and get high-quality reads, quality check (QC) (conducted on the basis of two criteria: max-missing (integrity parameter) <0.8 and Minor Allele Frequency (maf)> 0.01) and adapter trimming^9^ were performed. SNPs were discovered by aligning reads against a reference genome^68^ using STACKS v1.23 (parameters m3, M2 and N4). Genotyping was then done following steps reported in Jiao *et al*.^69^, excluding 3-bp positions, ambiguous reads, long homopolymer regions, and excessive numbers of low-quality positions (between 5 to 10).

The paired-end reads were merged by Pear software (Version 0.9.6)^70^. The merged reads were processed using a custom Perl script to trim adaptor sequences. The terminal 3-bp positions were also excluded from each read to eliminate artifacts that might have arisen from ligation sites. Reads with ambiguous bases (N) exceeding 8%, poor quality (15% nucleotide positions with a Phred quality <30), or without restriction sites were removed. The BsaXI tags in the genome of common carp were extracted based on the enzyme’s recognition site, which served as a reference for SNP discovery. High quality reads of each individual were aligned to the reference genome using SOAP2 (version 2.21)^71^ with the following parameters: *r* = 0, *M* = 4, *v* = 2. The aligned data for each individual were then used for SNP detection by RADtyping^72^ program with default parameters. For co-dominant markers, we used an ML algorithm to estimate for homozygotes or heterozygotes. In order to obtain robust results in the subsequent analyses, the following criteria were applied for SNP filtering. (1) Segregating markers that could be genotyped in at least 80% of the individuals were kept for analyses. (2) SNPs with a minor allele frequency (MAF) < 0.01 were discarded. (3) Polymorphic loci with more than two alleles possibly derived from sequencing or clustering errors were excluded. (4) Tags with more than two SNPs were excluded

### Genome-wide analysis and candidate SNP identification

Two programs were used to identify the candidate SNPs. Plink applies a general linear model and EMMAX applies a mixed linear model, which can control for the false positive rate. The BH method in p.adjust function in R was used to calculate the false discovery rate. Using Bonferroni correction (*P* < 0.05 and *P* < 0.01), −log10 (P value) was calculated and plotted on Manhattan graphs with 100,000 window size using Plink^73^, EMMAX^74^ and qqman package^75^ in R. In order to identify the candidate SNPs, two models (linear and mixed linear model) were considered. Plink applies the general linear model, and EMMAX applies the mixed linear model, which can control for the false positive rate. The BH method in p.adjust function in R language was used to calculate the false discovery rate. For each stage, we identified significant SNPs, and their chromosomal locations. After we selected the SNPs for these two stages, we filtered the common SNPs according to their positions on chromosomes in the reference genome.

EMMAX conducts GWAS analysis on the basis of variance component approach. The algorithm conducts association analysis of quantitative traits as described below:

Let n be the sample size, p the total number of genotyped SNPs and Y the vector of observed phenotypes. We used the genotype data to calculate the n x n matrix $\mathop{S}\limits^{\wedge }$ pairwise genetic relatedness between individuals, such as IBS or Balding-Nichols matrix, and normalized $\mathop{S}\limits^{\wedge }$ to have sample variance 1 using a Gower’s centered matrix. We also used BN matrix as recommended by the authors.

$${\mathop{S}\limits^{\wedge }}_{N}=\frac{({\rm{n}}-1)\mathop{S}\limits^{\wedge }}{{\rm{T}}{\rm{r}}(P\mathop{S}\limits^{\wedge }P)}$$

P = I-11'/n and 1 is vector of number one. $\mathop{S}\limits^{\wedge }$ is a positive-semidefinite matrix and can be replaced by other pairwise relatedness matrices estimated from the genotypes if they are also positive-semidefinite. Then we used a variance component model to estimate the restricted maximum likelihood parameters (or alternatively, maximum likelihood parameters) of ${\sigma }_{{\rm{a}}}^{2}$ and ${\sigma }_{{\rm{e}}}^{2}$ in

$${\rm{Var}}(Y)={\sigma }_{a}^{2}{\mathop{s}\limits^{\wedge }}_{N}+{\sigma }_{e}^{2}I$$

This tests the hypothesis H0: ${\sigma }_{{\rm{a}}}^{2}=0$. If the null hypothesis is rejected, it proceeds to step 3; otherwise, use the ordinary least squares to estimate the coefficients of each of the SNPs genotyped. For each marker, we used GLS F-test, or alternatively a score test, to estimate the effects ${\beta }_{k}$ and test the hypothesis ${\beta }_{k}\ne 0$ in the following model:

$${y}_{i}={\beta }_{0}+{\beta }_{k}{X}_{ik}+{\eta }_{i}\,{\rm{Var}}(\eta )={\rm{V}}\propto {\mathop{\sigma }\limits^{\wedge }}_{a}^{2}{\mathop{S}\limits^{\wedge }}_{N}+{\mathop{\sigma }\limits^{\wedge }}_{e}^{2}I$$

We substituted β_0_ by a multicolumn matrix containing the group information, as we thought that group information may be a confounding variable. We suspected that groups may cause population stratification (e.g. selection line and control line), which in turn may cause false positive signals in GWAS. β_0_ was accepted by –c parameter of EMMAX, which was a multicolumn file, such as:

100211 100211 1 1

100611 100611 1 1

100711 100711 1 0

100811 100811 1 0

101611 101611 1 1

101711 101711 1 1

First and second columns are sample names, third column is always 1, and fourth column is group information, with selected group coded as 1 and control group coded as 0.

In order to identify the candidate growth-associated genes, loci containing the selected SNP were annotated by SnpEff (version 4.1 g)^76^ and designated according to the published reference genome of the common carp (Songpu strain)^19^. Finally, plink and EMMAX results were merged by seeking a mathematic Union set. For each stage, we found significant SNPs, and identified their chromosome positions. After we selected the SNPs for these two stages, we filtered the common SNPs, i.e. the ones with corresponding relevant information (positions on chromosomes) and significant P values between the two stages. For these significant SNPs, we conducted LD analysis using Plink. The overlapping loci identified by these two methods were used to conduct the validation test. The common SNPs were used to conduct the linkage disequilibrium (LD) analysis, with ±0.5 M window along the chromosome or scaffold, using Plink and LDheatmap package^77^. Position name was constructed using chromosome or scaffold name plus position.

### Validation of growth performance markers

On the basis of the genomic region of DNA designated from the reference genome of common carp, specific primers for the genome-wide loci significantly associated with growth-related traits were designed using Primer3web 4.1.0 (for picking primers from DNA sequences) and Primer-BLAST (NCBI; for finding specific primers). PCR was conducted using 2×Es Taq MasterMix (CWBio, China), and results were checked on 1.8% agarose gels and verified on Agilent 2100 Bioanalyzer.

In total 180 individuals from the F3 generation of Xinlong carp strain (no relationship with the 2b-RAD sequence data) were randomly selected and used to validate the efficiency of the identified markers. After amplification of the SNPs of the three genes in these 188 individuals, three primer pairs were designed on the basis of corresponding sequences in the reference common carp genome (Table 5). PCR products were sequenced (one–way) and aligned using DNAMAN6.0 software.

**Table 5:** Specific primers designed for the loci overlapping between Plink and EMMAX analyses.

Scaffold	Reference sequence	Primers (5'-3')
NW_017538109.1–569817	Scaffold1618	F:TAGGATTGCCACTGCTGTAGACC R:GCTTCAGGATTTGCATCACTTTG
NW_017538165.1–106199	Scaffold28824	F: GCTGCTTGGCTGGATATACTGAA R:CAGTCAGCAGACACCAGTTCTCA
NW_017541427.1–19670	Scaffold9536	F:CAATGAAAGAGCCGTCAAATGTC R:AAGTGTCTCCCGATGAGGAAATC

Note: SNP name using scaffold or chrosomone name plus position

### Statistical data analysis

All data were represented as mean ± SE and subjected to analysis by Chi-square tests and Student Test using Statistical Package for Social Sciences (SPSS) for Windows (SPSS Inc., Chicago, IL, USA). Differences were considered statistically significant when P values were <0.05.

## Supplementary information

Supplementary Information.

## Data Availability

All of the 2b-RAD sequences used during this study are available from the NCBI Sequence Reads Archive (SRA) database under the accession numbers SRR6241620 and SRR6262716. The other datasets supporting the conclusions are included within the article and its additional files..
