# Supplementary material for: Genome Wide Analysis for Growth at Two Growth Stages in A New Fast-Growing Common Carp Strain (Cyprinus carpio L.)
Source: Sci Rep. 2020 Apr 29;10:7259. doi: 10.1038/s41598-020-64037-w (PMC7190712; doi:10.1038/s41598-020-64037-w)
Supplement: Supplementary file 1 — Supplementary Information. [file 41598_2020_64037_MOESM1_ESM.pdf]

Supplementary Information: Genome Wide Analysis for Growth at Two Growth Stages in A New Fast-Growing Common Carp Strain (*Cyprinus carpio* L.)

Shengyan Su [a, b, #, *], Bouzoualegh Raouf [a, b, #], Xinjin He [a, c], Nana Cai [a], Xinyuan Li [b], Juhua Yu [b], JianLin Li [b], Fan Yu [b], Meiyao Wang [b], Yongkai Tang [a, b, *].

[a] Key Laboratory of Freshwater Fisheries and Germplasm Resources Utilization, Ministry of Agriculture; Freshwater Fisheries Research Center, Chinese Academy of Fishery Sciences, Wuxi 214081, PR China.
[b] Wuxi Fisheries College, Nanjing Agricultural University, Wuxi 214081, PR China.
[c] College of Animal science, Shanxi Agricultural University, Taigu, PR China.
[#] These authors contributed equally to this work.
[*] Corresponding author:
Shengyan Su
susy@ffrc.cn
Tel.: +83-510-85555796
Yongkai Tang
tangyk@ffrc.cn

Supplementary Table S1: Summary of the constructed tags for each of the 123 specimens after digestion with BsaXI Restriction Enzyme site.

Sample	Enzyme	Tag number	Depth	Mapping rate
4395	20,020,774	195,696	48.07	46.99%
4505	16,765,336	190,000	41.59	47.13%
4518	19,571,517	199,866	46.57	47.56%
4282	17,143,844	200,198	41.01	47.89%
4363	16,373,663	206,161	38.07	47.93%
4299	20,904,750	209,633	47.87	48.01%
4224	17,279,232	205,065	40.46	48.02%
4291	18,088,765	206,246	42.16	48.08%
4514	21,549,011	201,381	51.44	48.08%
4397	22,618,716	222,494	49.04	48.24%
4296	20,868,195	210,591	47.92	48.36%
4394	19,946,278	210,796	45.76	48.36%
4434	20,062,865	225,156	43.10	48.37%
4289	17,976,975	206,394	42.17	48.41%
4179	20,096,399	222,332	43.80	48.45%
4513	21,669,879	210,034	50.00	48.46%
4272	17,221,395	202,730	41.29	48.61%
4183	17,382,997	218,050	38.76	48.62%
4163	17,288,864	214,720	39.21	48.70%
4390	14,557,242	184,266	38.51	48.74%
4150	16,076,844	215,030	36.44	48.74%
4165	24,584,168	238,701	50.20	48.75%
4286	17,879,482	231,878	37.62	48.79%
4192	22,186,397	221,692	48.83	48.79%
4102	27,502,863	227,333	59.04	48.80%
4245	15,546,475	204,550	37.09	48.80%
4114	26,734,249	228,485	57.12	48.82%
4138	17,437,641	216,059	39.42	48.84%
4243	15785070	199443	38.66268057	0.488499639
4070	25406030	239535	51.87064521	0.489050631
4078	26627017	236453	55.08700249	0.489183111
4180	21985718	238006	45.26690504	0.490036077
4255	19801263	205883	47.16152378	0.490360438
4223	17250302	234224	36.15851066	0.490958999
4325	21913992	238068	45.21206126	0.491172261
4364	22762894	235063	47.57199134	0.491256296
4115	26734968	227330	57.80585932	0.491528772
4432	30810481	240845	62.92882975	0.49191358
4321	19073726	233160	40.26532853	0.492209231

4104	27586530	228164	59.65352553	0.49338525
4506	19720082	236229	41.22215308	0.49380464
4358	21147832	237077	44.06018298	0.493935076
4131	23042061	224016	50.80694683	0.493947525
4234	19499231	238292	40.49984892	0.494931826
4327	19209242	234356	40.57339262	0.495002249
4392	25359941	239406	52.48553086	0.495480293
4111	26684750	239572	55.24234886	0.495958178
4124	24462565	227013	53.52870981	0.496747295
4419	17466059	214188	40.51478141	0.496836751
4094	21496237	227237	47.04798074	0.497344814
4276	17139673	232682	36.65861992	0.497664162
4083	21328323	237985	44.60901317	0.49775484
4240	21491532	235737	45.3860828	0.497832309
4387	22683427	228038	49.5314509	0.49794297
4295	20842063	235901	44.05373441	0.498622425
4284	19107077	235892	40.47039323	0.49963906
4372	16790631	231436	36.30888885	0.500468624
4336	14882703	233687	31.88627951	0.500675784
4093	21810016	237450	46.00792167	0.500897432
4516	19726581	234271	42.34363195	0.502868946
4112	26738695	239421	56.52017576	0.506087414
4409	20198909	233902	43.89800429	0.508335921
4339	14887881	201468	34.64847519	0.468875255
4375	16726373	191213	41.59891848	0.475551633
4389	14675726	199642	35.23267649	0.479289542
4420	17625277	177807	47.54650829	0.479657823
4263	17816083	200538	42.98180395	0.483803595
4162	17261001	220918	37.87453716	0.484744019
4401	15902580	214534	35.97500163	0.485321313
4265	17793868	202819	42.74265232	0.487191543
4433	20040402	225723	43.29353234	0.487632234
4201	25968913	240101	52.7485225	0.487697464
4517	19765223	211927	45.57657118	0.488681863
4149	16135510	215707	36.57326373	0.488928395
4184	28973793	241543	58.69231151	0.489294481
4196	26593131	241167	53.98414377	0.489569807
4370	17041431	205462	40.64035685	0.489985201
4144	22196310	237623	45.78419177	0.490143497
4366	20838085	236410	43.21111205	0.490234059
4157	18408831	219943	41.03487722	0.490271979
4283	19144334	232377	40.40164044	0.490401599
4159	23076929	240246	47.12903857	0.490644271

4320	18676548	220236	41.60965056	0.49066578
4132	21635224	238609	44.50061817	0.490785212
4379	14289154	200286	35.04449138	0.491206197
4515	24419150	238893	50.25868485	0.491681651
4343	19455965	234052	40.92049203	0.492266665
4275	17004919	229788	36.4360454	0.492361416
4333	19062520	224954	41.73206967	0.492474028
4089	21371755	240034	43.85215844	0.492519636
4099	25244548	226961	54.8089804	0.492759902
4266	17055726	229953	36.5538784	0.492835896
4247	19657675	231280	41.95655483	0.493634776
4226	20041667	223824	44.21672385	0.493809422
4411	19938304	232722	42.3600648	0.494431171
4107	25989306	239275	53.71477589	0.49453429
4147	18283785	231034	39.17924634	0.495069156
4427	25370345	238979	52.56999987	0.495189403
4097	25215468	227644	54.88757446	0.495522312
4077	26652745	241073	54.82253093	0.495867574
4241	21353553	226226	46.8187211	0.496011694
4090	21450589	239874	44.3964373	0.496468931
4100	27231518	239789	56.40190751	0.496650866
4288	18005126	233885	38.25625842	0.496945425
4329	17421977	235905	36.72551663	0.497287593
4512	21641073	235285	45.74300954	0.497324878
4378	14352269	226899	31.46860057	0.497495831
4442	18772256	234096	39.91167726	0.497711303
4261	17872425	233073	38.24432259	0.498741441
4507	15521199	229685	33.70452141	0.498764496
4203	20363652	237195	42.82161091	0.498784403
4237	15727861	231529	33.90691879	0.499141937
4285	18711998	233301	40.0878436	0.499814825
4117	24423025	238000	51.31128571	0.500023482
4421	21329515	239818	44.48300378	0.500143815
4193	20106799	233300	43.16704243	0.500868935
4359	16387814	236053	34.81714276	0.501512343
4160	17183634	235214	36.66883349	0.50193242
4101	27504159	239325	57.93743654	0.504137465
4198	20410168	237242	43.38501614	0.504295114
4110	26082221	239593	54.90253054	0.504338262
4118	24546694	238270	52.12529903	0.505970173
4393	19873020	234678	42.94504385	0.507132635
	20,474,874			

Supplementary Table S2: Annotation for all SNPs called using reference genome of common carp.

Type (alphabetical order)	Count	Percent
_prime_UTR_variant	3142	0.01215
5_prime_UTR_premature_start_codon_gain_variant	382	0.00148
5_prime_UTR_variant	2110	0.00816
downstream_gene_variant	34085	0.13182
intergenic_region	70276	0.27179
intragenic_variant	2992	0.01157
intron_variant	87908	0.33998
missense_variant	7034	0.0272
missense_variant+splice_region_variant	140	0.00054
non_coding_exon_variant	5079	0.01964
splice_acceptor_variant+intron_variant	26	0.0001
splice_acceptor_variant+splice_donor_variant+intron_variant	3	0.00001
splice_donor_variant+intron_variant	30	0.00012
splice_region_variant	33	0.00013
splice_region_variant+intron_variant	1254	0.00485
splice_region_variant+non_coding_exon_variant	99	0.00038
splice_region_variant+stop_retained_variant	1	0
splice_region_variant+synonymous_variant	172	0.00067
start_lost	12	0.00005
stop_gained	84	0.00032
stop_gained+splice_region_variant	3	0.00001
stop_lost	11	0.00004
stop_lost+splice_region_variant	1	0
stop_retained_variant	4	0.00002
synonymous_variant	9057	0.03503
transcript	70	0.00027
upstream_gene_variant	34559	0.13366

Type (alphabetical order)	Count	Percent
DOWNSTREAM	34085	0.13182
EXON	21413	0.08281
INTERGENIC	70276	0.27179
INTRON	87908	0.33998
NONE	3062	0.01184
SPLICE_SITE_ACCEPTOR	29	0.00011
SPLICE_SITE_DONOR	30	0.00012
SPLICE_SITE_REGION	1559	0.00603
TRANSCRIPT	12	0.00005

UPSTREAM	34559	0.13366
UTR_3_PRIME	3142	0.01215
UTR_5_PRIME	2492	0.00964

**Type (alphabetical order)**	**Count**	**Percent**
MISSENSE	7198	0.43574
NONSENSE	87	0.00527
SILENT	9234	0.55899

**Transitions**	7109588
**Transversions**	5149691
**Ts/Tv ratio**	1.3806

Blen5m

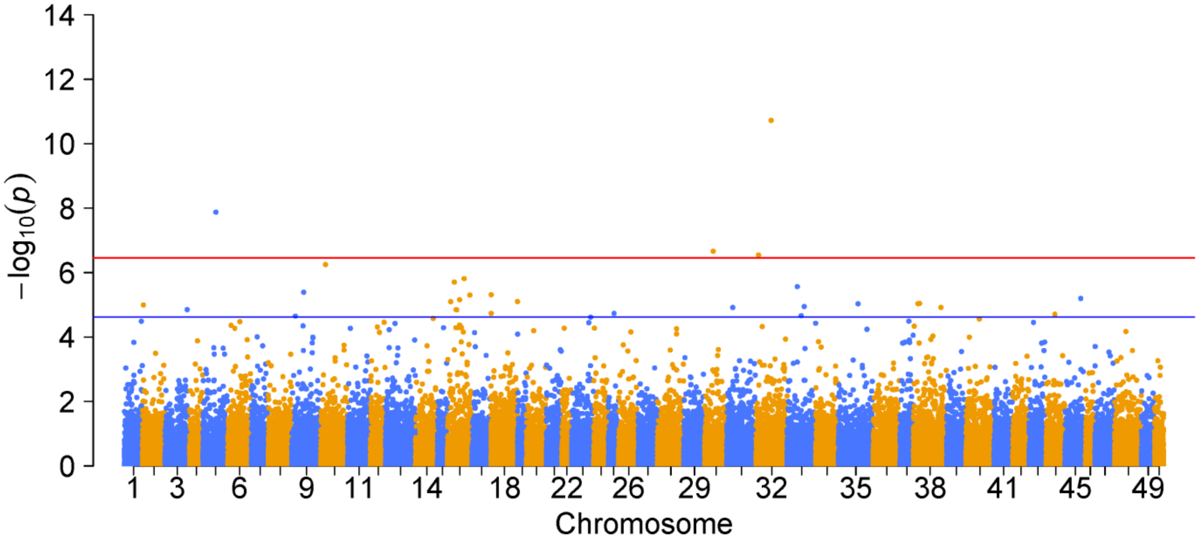

Figure S1. Manhattan plots for Blen5m (NW_017538109.1-569817 and NW_017541427.1-19670)

Bwid5m

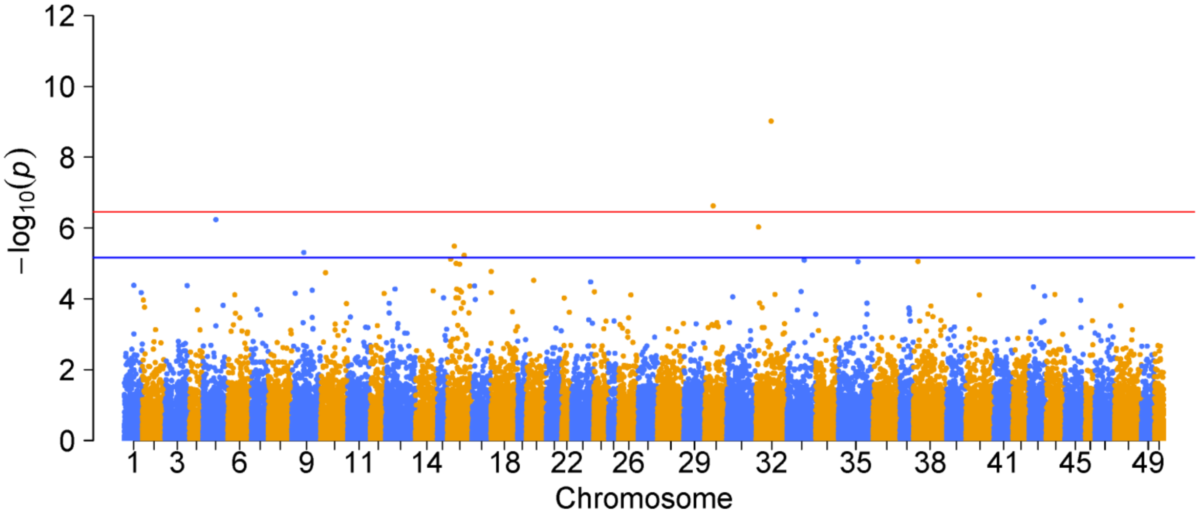

Figure S2. Manhattan plots for Bwid5m (W_017538165.1-106199 and NW_017541427.1-19670)

Linkage disequilibrium for the three SNPs

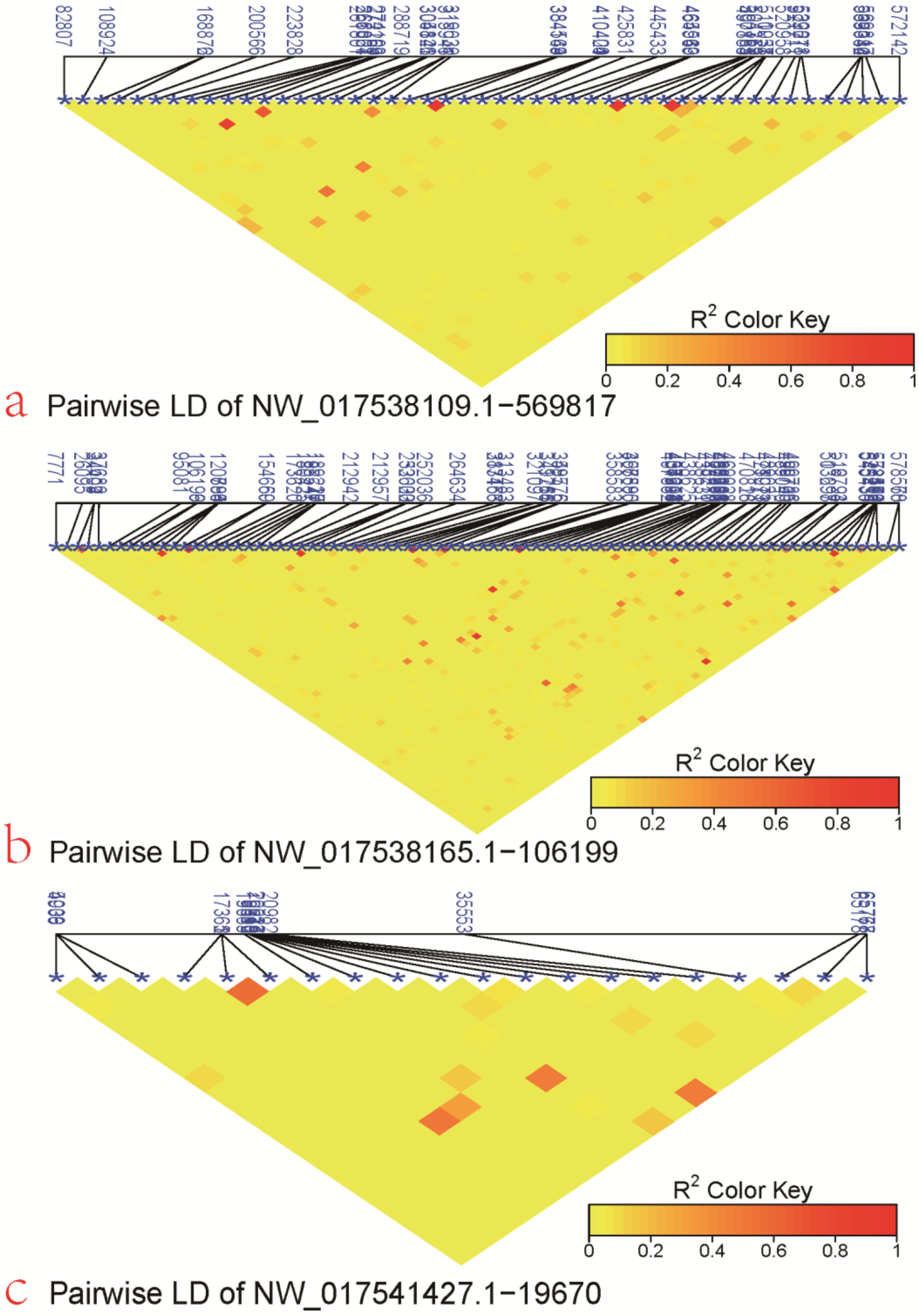

Figure S3. LDheatmap for the common loci explored by Plink and EMMAX. a. LDheatmap for NW_017538109.1-569817. b. LDheatmap for NW_017538165.1-106199. c. LD heatmap for NW_017541427.1-19670.
